# N-acetyl cysteine attenuates oxidative stress and glutathione-dependent redox imbalance caused by high glucose/high palmitic acid treatment in pancreatic Rin-5F cells

**DOI:** 10.1371/journal.pone.0226696

**Published:** 2019-12-20

**Authors:** Arwa Alnahdi, Annie John, Haider Raza

**Affiliations:** Department of Biochemistry, College of Medicine and Health Sciences, United Arab Emirates University, Al Ain, United Arab Emirates; University of Texas Medical Branch at Galveston, UNITED STATES

## Abstract

Elevated levels of glucose and fatty acids are the main characteristics of diabetes, obesity and other metabolic disorders, associated with increased oxidative stress, mitochondrial dysfunction and inflammation. Once the primary pathogenesis of diabetes is established, which is potentially linked to both genetic and environmental factors, hyperglycemia and hyperlipidemia exert further destructive and/or toxic effects on β-cells. The concept of glucolipotoxicity has arisen from the combination of deleterious effects of chronic elevation of glucose and fatty acid levels on pancreatic β- cell function and/or survival. Though numerous studies have been conducted in this field, the exact molecular mechanisms and causative factors still need to be established. The aim of the present work was to elucidate the molecular mechanisms of oxidative stress, and inflammatory/antioxidant responses in the presence of high concentrations of glucose/fatty acids in a cell-culture system using an insulin-secreting pancreatic β-cell line (Rin-5F) and to study the effects of the antioxidant, N-acetyl cysteine (NAC) on β-cell toxicity. In our study, we investigated the molecular mechanism of cytotoxicity in the presence of high glucose (up to 25 mM) and high palmitic acid (up to 0.3 mM) on Rin-5F cells. Our results suggest that the cellular and molecular mechanisms underlying β-cell toxicity are mediated by increased oxidative stress, imbalance of redox homeostasis, glutathione (GSH) metabolism and alterations in inflammatory responses. Pre-treatment with NAC attenuated oxidative stress and alterations in GSH metabolism associated with β-cells cytotoxicity.

## Introduction

Glucose and fatty acids are the main sources of energy production and cell survival. However, overload of these nutrients, has been implicated in diabetes and obesity-induced metabolic reprogramming and complications as also in cardiovascular disorders and cancer [1–5]. However, the specific pathogenesis of these diseases remains unclear. Glucotoxicity and lipotoxicity caused by chronic hyperglycemia/dyslipidemia have been proposed to play a critical role in disease development [2, 4, 6, 7, 8]. Persistent hyperglycemia reduces β-cell function and insulin action by attenuation of insulin-mediated glucose transport and impairment of glucose-induced insulin secretion, which subsequently leads to deterioration of β-cell function. In addition, excessive exposure to high levels of fatty acids causes β-cell dysfunction, inhibits glucose-induced insulin secretion, and induces β-cell death by apoptosis [9]. The combination of glucolipotoxicity exacerbates the deleterious effects of chronic elevation of glucose and fatty acids on pancreatic β-cell function and/or survival [10,11]. Studies have shown that elevated glucose levels augment the effect of free fatty acid (FFA)-induced cell death, because high glucose concentration inhibits fat oxidation, and consequently lipid detoxification [12]. We have recently demonstrated that HepG2 cells treated with a high (25 mM) glucose concentration induces glucotoxicity and metabolic stress, which is further augmented by the treatment of saturated fatty acids [13].

Though numerous studies have been carried out in this field, the exact molecular mechanisms and causative factors involved in glucolipotoxicity is not clearly understood. This is due to the fact that under *in vivo* conditions, several physiological, physical, endocrine, dietary and environmental factors work in tandem. Therefore, our aim in the present study was to elucidate the molecular and cellular mechanisms underlying pancreatic β-cell toxicity in the presence of high glucose/ palmitic acid using an *in vitro* model of insulin-secreting pancreatic cells, Rin-5F. The main focus in this study was to investigate the oxidative stress induced, changes in redox homeostasis, GSH metabolism and inflammatory responses in pancreatic β-cells after treatment with high levels of glucose and, palmitic acid. Furthermore, we also investigated the effects of N-acetyl cysteine (NAC), a reactive oxygen species (ROS) scavenger, on the modulation of oxidative stress and inflammation in glucolipotoxicity-induced cells. Our results indicate that NAC pre-treatment selectively restores redox homeostasis, while exerting a marginal effect on the inflammation induced alterations in these cells.

## Materials and methods

### Materials

Reduced and oxidized glutathione (GSH/GSSG), 1-chloro 2, 4-dinitrobenzene (CDNB), cumene hydroperoxide, glutathione reductase, 3-(4,5-dimethylthiazol-2-yl)-2,5-diphenyltetrazolium bromide (MTT), NADH, NADPH, LookOut mycoplasma PCR detection kit, fatty acid-free bovine serum albumin (BSA), palmitic acid and N-acetyl cysteine (NAC) were purchased from Sigma (St Louis, MO, USA), while 2',7'-dichlorofluorescein diacetate (DCFDA) was procured from Molecular Probes (Eugene, OR, USA). Kits for nitric oxide (NO) were purchased from R & D Systems (MN, USA) and that for lipid peroxidation (LPO) from Oxis Int, Inc. (Portland, OR, USA). Kits for GSH/GSSG assays were procured from Promega Corp. (Madison, WI, USA). Tumor necrosis factor alpha (TNF-α) and interleukin 6 (IL-6) kits were purchased from BD Pharmingen (BD Biosciences, San Jose, USA) and kits for prostaglandin E2 (PGE2) were purchased from Abcam (Cambridge, UK). Kits for catalase were purchased from Cayman (MI, USA) while those for superoxide dismutase (SOD) were purchased from Trevigen (Gaithersburg, MD, USA). Rin-5F cells were obtained from the American Type Culture Collection (Manassas, VA, USA). The Rin-5F cell line, a commonly used *in vitro* model for insulin secreting cells, is a clone derived from the Rin-m rat pancreatic islet cell line. Polyclonal antibodies against NF-κBp65 and actin were purchased from Santa Cruz Biotechnology Inc. (Santa Cruz, CA, USA). Reagents for cell culture, SDS-PAGE and Western blot analyses were purchased from Gibco BRL (Grand Island, NY, USA) and Bio Rad Laboratories (Richmond, CA, USA).

### Cell culture and treatment

Rin-5F cells were cultured in RPMI 1640 with glutaMAX medium supplemented with 10% heat inactivated FBS and 1% non-essential amino acids in a humidified incubator in the presence of 5% CO_2_-95% air at 37°C. The cell line was tested for mycoplasma contamination using the LookOut mycoplasma PCR detection kit (Sigma, St Louis, MO, USA) and tested negative (S1 Fig). Palmitic acid was dissolved in 100% ethanol and heated to 40°C for 10 min to make a stock solution of 100 mM and then conjugated to 1% fatty acid- free BSA in a molar ratio of 6:1, according to the method described in previously published reports [14, 15] The palmitate/BSA conjugate was added to cultured cells in RPMI supplemented with 1% FBS to generate a final concentration of 0.06 mM and 0.3 mM palmitate. For high glucose concentration, similar treatment was carried out in the presence of high glucose (25 mM) media (to mimic the in vivo diabetic condition). To normalize the effect of BSA/ethanol used in palmitic acid treatment, control cells in both normal and high glucose media, were treated with medium containing equivalent amounts of vehicle (BSA/ethanol), in the absence of palmitic acid. For NAC treatment, cells were treated with 10 mM NAC 2 h prior to the palmitic acid treatment. Concentrations of glucose and palmitic acid and time points used in the study, were based on cytotoxicity tests and numerous previously published reports [14,15]. These concentrations were used to mimic the human hyperglycemia/hyperlipidemia under experimental *in vitro* conditions [14–15]. After the required treatments, harvested cells were washed twice with cold phosphate buffered saline (PBS, pH 7.4) and homogenized in H-medium buffer (70 mM sucrose, 220 mM mannitol, 2.5 mM HEPES, 2 mM EDTA, 0.1 mM phenylmethylsulfonylfluoride, pH 7.4) at 4°C, to prepare total cell lysates. Cellular fractionation to prepare nuclear and cytosolic extracts were performed by centrifugation and the purity of isolated fractions for cross contamination was checked as described previously [16]. Bradford method was used to determine protein concentrations of the lysates [17].

### MTT cell viability assay

The mitochondria dehydrogenase activities were determined by MTT assay. Briefly, cells were treated with different concentrations of palmitic acid (0.02–0.5 mM) for different time intervals (2–48 h) under normal and high glucose conditions. The cell viability was tested after treatment and assessed by the reduction of MTT dye to form insoluble purple formazan crystals. The crystals were dissolved in acidified alcohol, and the viable cells were quantitated using an ELISA reader (TECAN Infinite M 200 PRO, Austria) at 550 nm after subtracting the appropriate control values.

### Measurement of ROS, NO and LPO

Intracellular production of peroxides were measured fluorometrically using the DCFDA-dependent fluorescence method. This compound by itself is not fluorescent, it and is converted by the intracellular esterases to 2,7’-dichlorodihydrofluorescin which is subsequently oxidized by hydrogen peroxide to the highly fluorescent 2,7’-dichlorodihydrofluorescein (DCF) and ROS production was then measured microscopically and fluorometrically as described previously[18]. The measurement of ROS production was also done using FACS analysis. For this, cells were incubated with 5μM DCFDA for 30min at 37°C, the cells were then washed with PBS, trypsinized, resuspended in PBS and fluorescence analyzed immediately by flow cytometry as described previously[19]. For the analysis of the flow cytometry data, the untreated cells were used as the negative control, and the statistical tool in the software was used to calculate the percentage fluorescence in the experimental cells.

For the NO assay, NO production was determined based on nitric oxide synthase (NOS) activity by measuring the concentration of total nitrite in culture supernatants using Griess reagent (R & D Systems Inc.).

The LPO in cell extracts was measured using the LPO-586 kit according to the manufacturer’s recommended protocol and the concentration of malonedialdehyde (MDA) calculated from the standard curve using MDA as standard [20].

### Measurements of SOD and catalase activity

Measurement of catalase was based on its peroxidatic activity, which depends on its ability to catalyze the oxidation of alcohol by hydrogen peroxide. The formaldehyde produced was measured colorimetrically with a chromogen, Purpald, which on oxidation, changes from colorless to purple, and the absorbance read at 540 nm.

The SOD assay was based on the conversion of xanthine to uric acid and hydrogen peroxide by xanthine oxidase. Superoxide ions reduced the nitro blue tetrazolium (NBT, yellow water soluble) to NBT-diformazan (dark-blue water insoluble). SOD activity was measured as the percent inhibition in NBT-diformazan formation, according to the vendor's protocol (R &D System, MN, USA).

### Measurements of GSH and GSH metabolism

The GSH/GGSG ratio was measured using the GSH/GGSG-Glo kit as per the vendor’s protocol. The activity of glutathione S-transferase (GST), using CDNB as substrate, was measured as described by Habig *et al*. [21]. The activity of GSH reductase, using oxidized glutathione (GSSG) as substrate, was measured by a standard protocol by Smith *et al*. [22]. Glutathione peroxidase (GSH-Px) activity was measured indirectly by a coupled reaction with glutathione reductase using cumene hydroperoxide as a substrate as described previously[23].

### Measurement of cytokines (TNF-α, IL-6 and PGE2)

TNF-α, IL-6 and PGE2 were measured in culture supernatants using specific ELISA kits. These kits were procured from BD Pharmingen (BD Biosciences, San Jose, USA) for TNF-α and IL-6 assays and from Abcam (Cambridge, UK) for the PGE2 assay. The cytokines were measured as described in the vendor’s protocols [19].

### Measurement of protein expression

Proteins from cell extracts (30 μg) were resolved by 7.5% SDS-PAGE and electrophoretically transferred on to nitrocellulose membranes by Western Blotting, as described [18,24,25]. The blots were then developed using an ECL Plus Western Blotting Luminol Reagent kit and the bands visualized using the Typhoon FLA 9500 system (GE Healthcare, Uppsala, Sweden). Densitometric analysis was performed using Image J software and expressed as relative ratio normalized against the loading control, β-actin.

### Statistical analysis

Values shown are expressed as the mean ± S.E.M. of three individual experiments. Statistical significance of the data was assessed using SPSS software (version 23) by analysis of variance (ANOVA) followed by least significant difference (LSD) post-hoc analysis. P values ≤ 0.05 were considered statistically significant.

## Results

### Effect of high glucose/high palmitic acid treatment on Rin-5F cell viability

**Fig 1:**
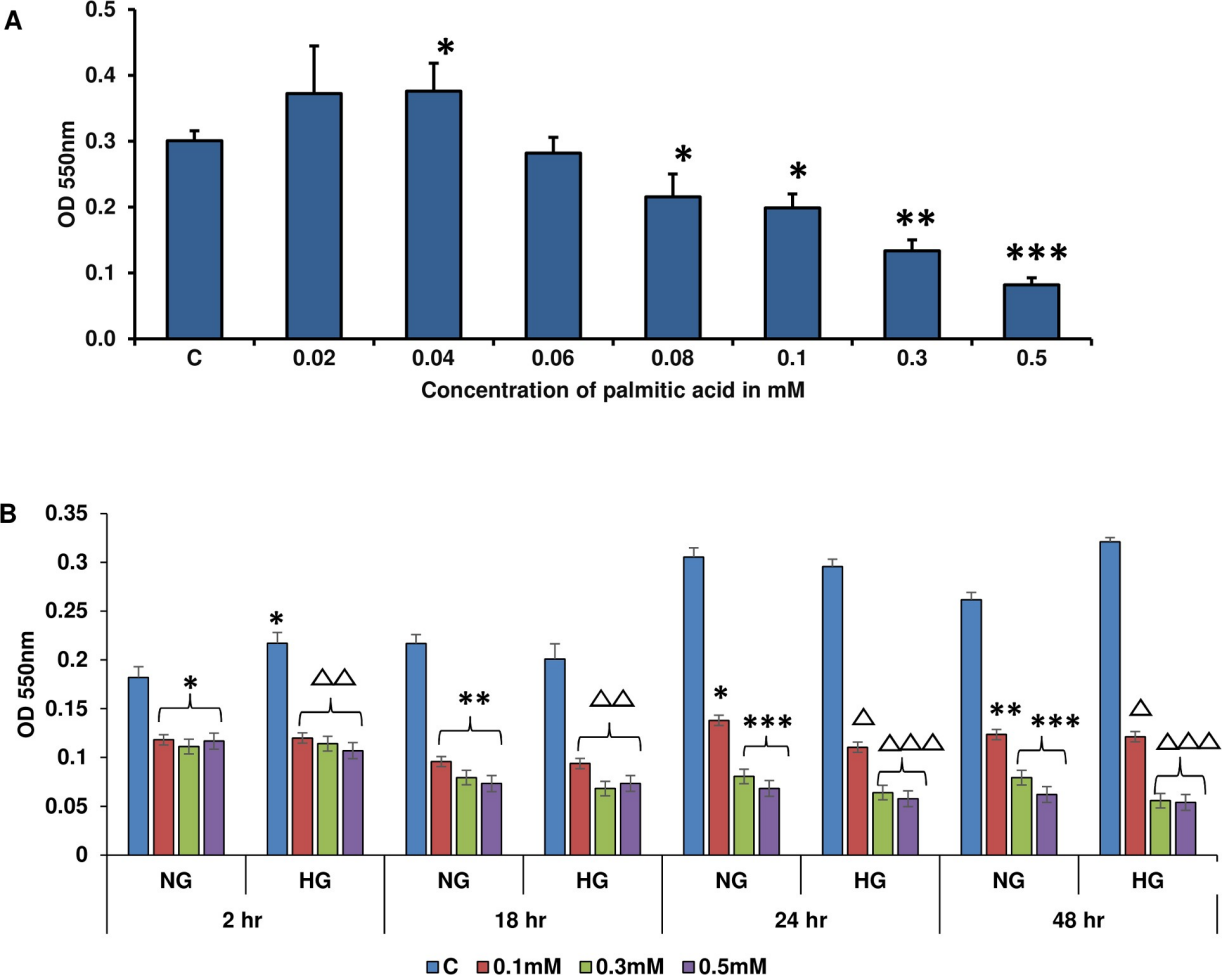
MTT cell viability assays after treatment with palmitic acid under normal and high glucose conditions. Rin-5F cells were grown in 96-well plates. Cells were treated with: (A) different concentrations of palmitic acid (0.02–0.5 mM) under normal glucose conditions for 24 h, (B) different concentrations of palmitic acid (0.1–0.5 mM) for different time intervals (2–48 h) under normal and high glucose conditions. Results are expressed as the mean +/- S.E.M. of three experiments. Asterisks indicate significant differences (*p ≤ 0.05, **p ≤ 0.01, ***p ≤ 0.001) relative to untreated control cells under normal glucose conditions, and triangles indicate significant differences (Δp ≤ 0.05, ΔΔp ≤ 0.01, ΔΔΔp ≤ 0.001) relative to untreated control cells under high glucose conditions.

Fig 1A shows the inhibition of cell survival in the presence of high glucose/high palmitic acid. Maximum inhibition was observed in cells treated with 0.3–0.5 mM palmitic acid (~ 56% and 74%, respectively). Negligible toxicity was observed using palmitic acid at concentrations below 0.06 mM. However, cells treated with 0.08 mM or 0.1 mM palmitic acid exhibited an inhibition of cell survival by 30% and 36%, respectively. Fig 1B shows the time-dependent inhibition of cell survival in the presence of normal and high glucose. Maximum inhibition of cell viability was observed with 0.3–0.5 mM palmitic acid at 24–48 h. For further studies, we selected 0.06 mM and 0.3 mM palmitic acid concentrations, since 0.5 mM was found to be extremely toxic with low yield of cells.

### Effect of high glucose/high palmitic acid treatment on oxidative stress

**Fig 2:**
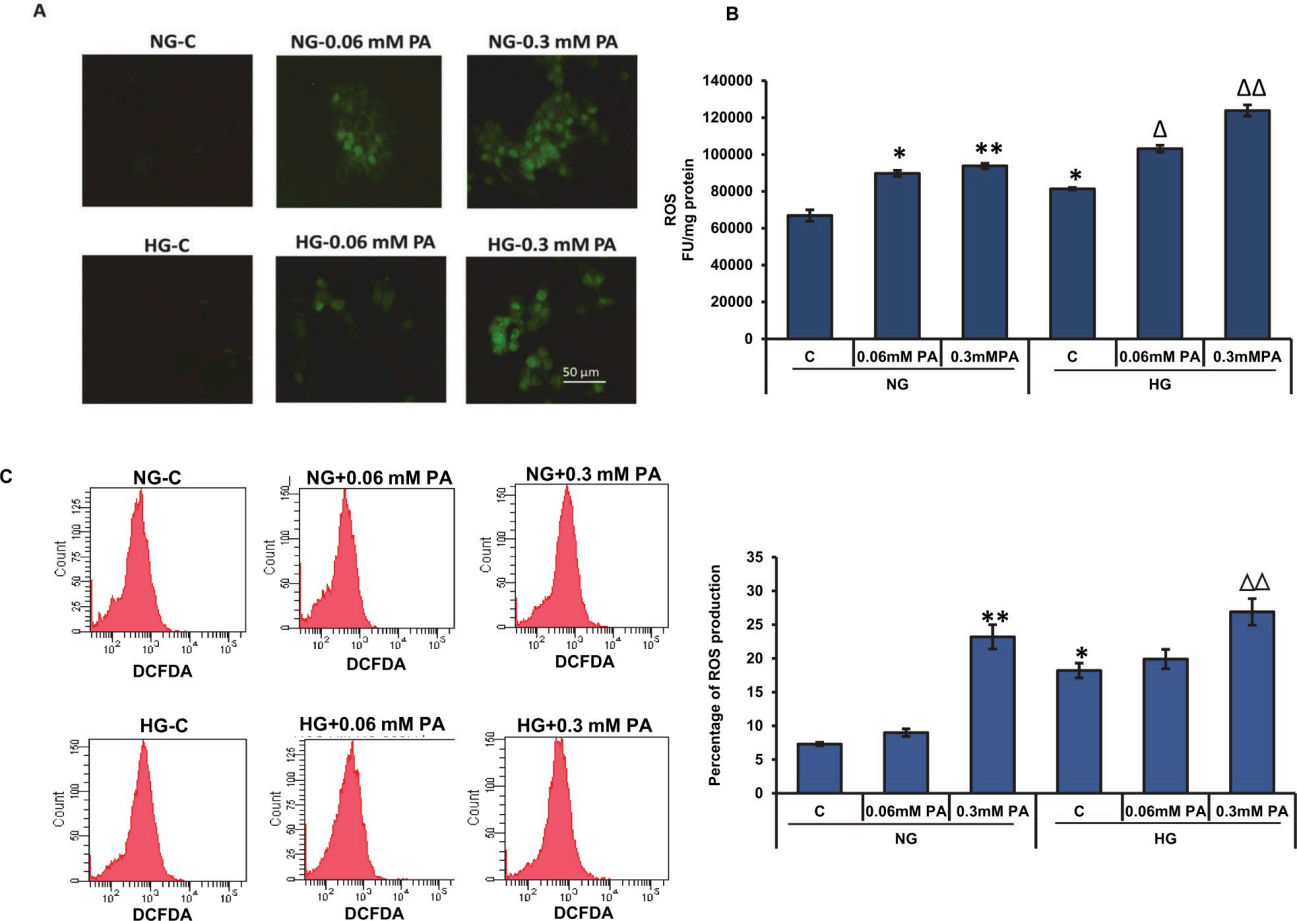
High glucose/high palmitic acid-induced ROS production. Intracellular production of ROS was measured in Rin-5F cells treated with different concentrations of palmitic acid under normal and high glucose conditions using the cell permeable probe, DCFDA (A). Cells were grown on cover slips and incubated with 5 μM DCFDA for 30 min at 37°C. Cells were washed twice with PBS and fluorescence was immediately visualized using an Olympus fluorescence microscope. Original magnification ×200. Production of ROS was also measured fluorometrically (B) and by flow cytometry (C) using FACSDiva software as described in the Materials and Methods. Results are expressed as the mean +/- S.E.M. of three experiments. Asterisks indicate significant differences (*p ≤ 0.05, **p ≤ 0.01, ***p ≤ 0.001) relative to untreated control cells under normal glucose conditions, and triangles indicate significant differences (Δp ≤ 0.05, ΔΔp ≤ 0.01, ΔΔΔp ≤ 0.001) relative to untreated control cells under high glucose conditions.

Treatment with palmitic acid showed a concentration-dependent increase in intracellular ROS production under normal and high glucose conditions. Reactive oxygen species was measured and captured microscopically using DCFDA. Maximum fluorescence was observed with high concentration of palmitic acid (0.3 mM) under both normal as well as high glucose conditions (Fig 2A). Intracellular ROS production was also measured spectrofluorometrically (Fig 2B) and by flow cytometry using the BD FACSDiva software (Fig 2C). The higher concentration of palmitic acid (0.3 mM) showed a marked increase in ROS production (5-fold and 1.5-fold compared with the control of normal and high glucose conditions, respectively).

**Fig 3:**
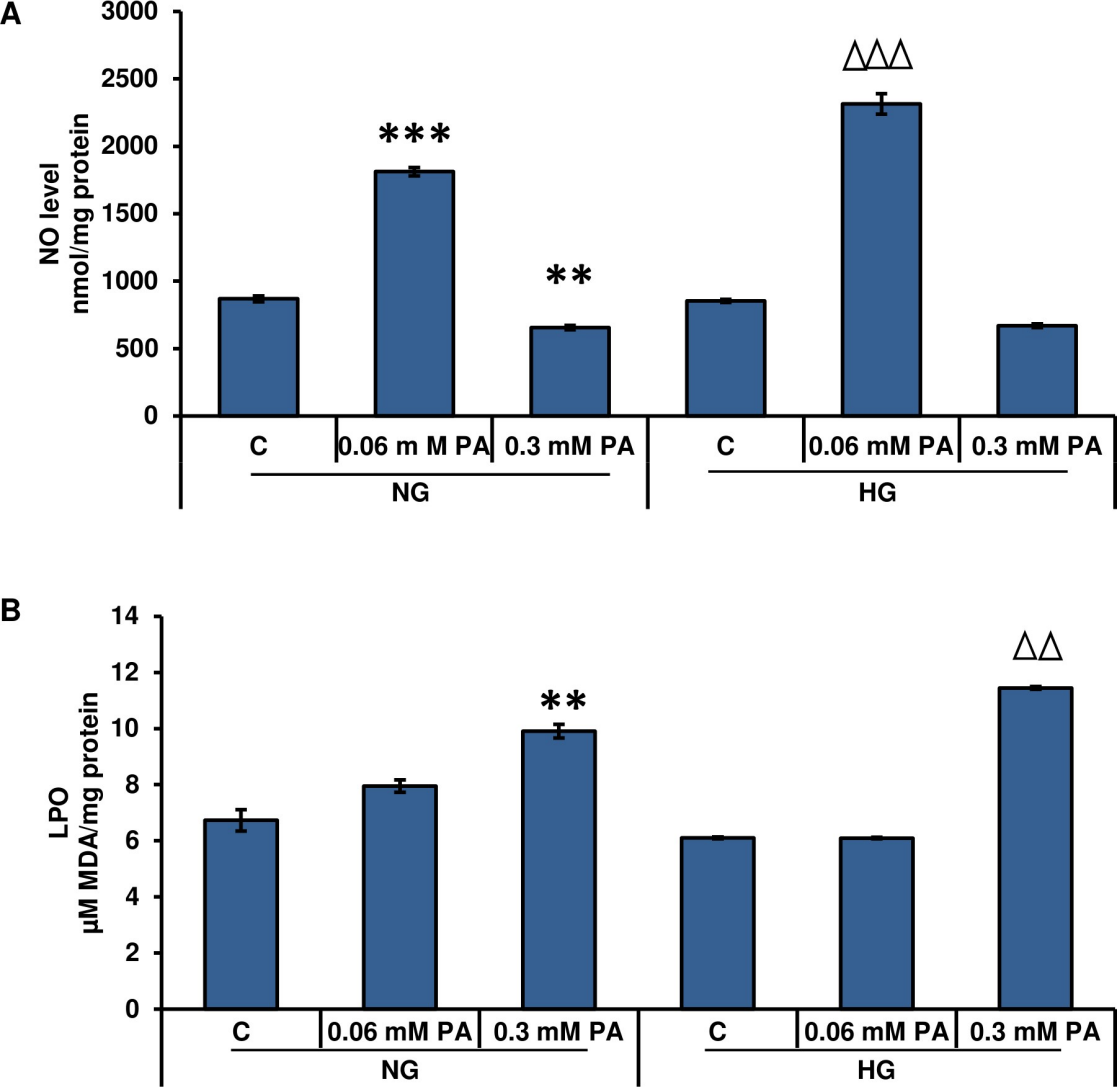
High glucose/high palmitic acid-induced NO production and lipid peroxidation in Rin-5F cells. NO production was determined by measuring the concentration of total nitrite in culture supernatants (A) with Griess reagent. Lipid peroxidation (LPO) was measured as the total amount of malonedialdehyde (B) as per the vendor’s protocol. Results are expressed as the mean +/- S.E.M. of three experiments. Asterisks indicate significant differences (**p ≤ 0.01, ***p ≤ 0.001) relative to untreated control cells under normal glucose conditions, and triangles indicate significant differences (ΔΔp ≤ 0.01, ΔΔΔp ≤ 0.001) relative to untreated control cells under high glucose conditions.

Nitric oxide production was significantly increased (~ 50%) in Rin-5F cells treated with 0.06 mM palmitic acid under normal and high glucose conditions (Fig 3A). In contrast, 0.3 mM palmitic acid showed a slight reduction in NO production.

In parallel with ROS production, LPO was significantly increased in a concentration-dependent manner after treatment with palmitic acid under normal and high glucose conditions (Fig 3B). Treatment with 0.3 mM palmitic acid under high glucose conditions caused a marked increase (~2-fold) in MDA production.

### Effect of high glucose/high palmitic acid treatment on SOD and catalase activity

**Fig 4:**
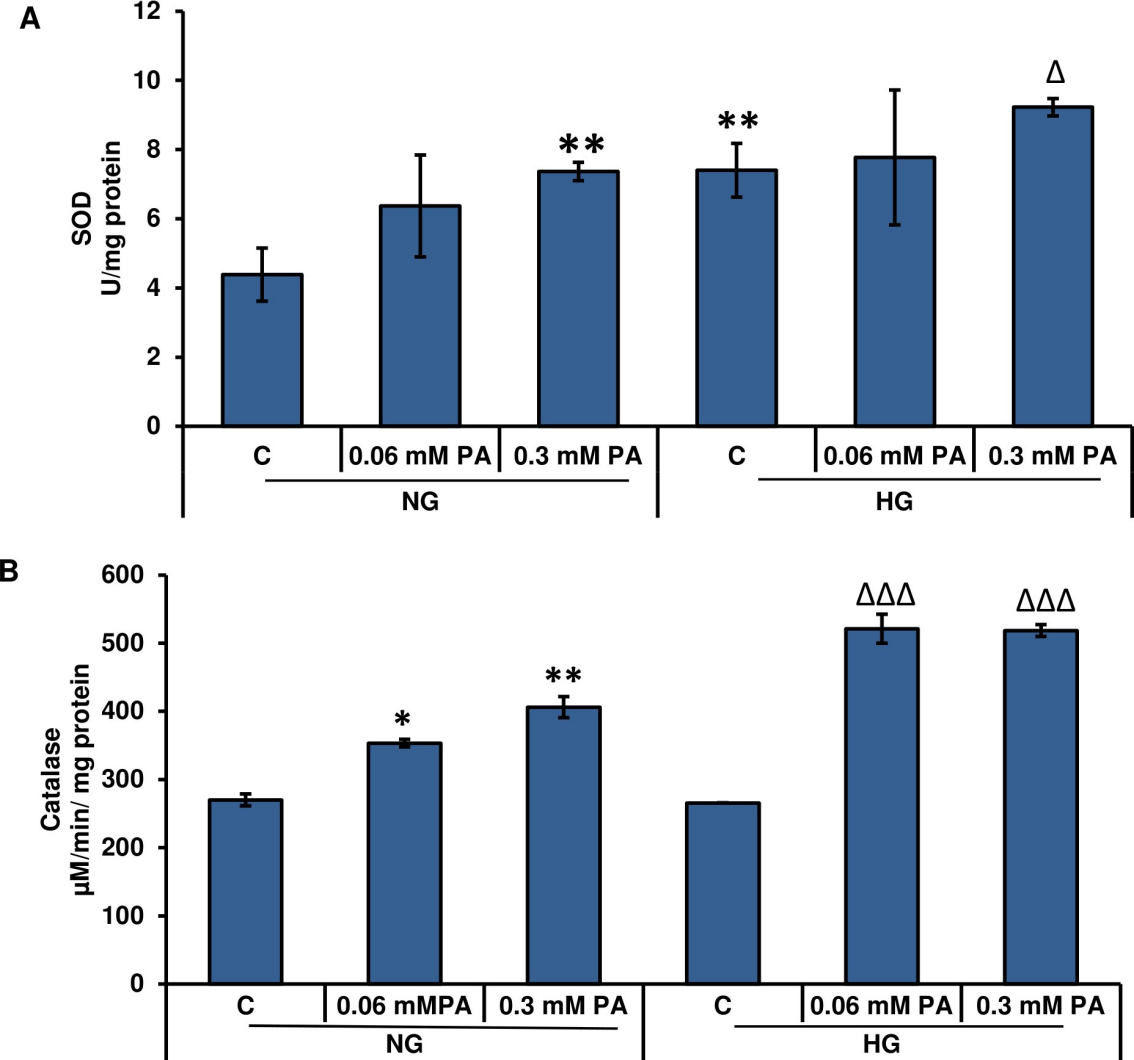
High glucose/high palmitic acid treatment-induced alterations in the superoxide dismutase (SOD) and catalase enzyme activities. Rin-5F cells were treated with different concentrations of palmitic acid under normal and high glucose conditions as described in Materials and Methods. SOD (A) was measured as the percentage conversion of nitro blue tetrazolium (NBT) to NBT-diformazan according to the vendor’s protocol. The percentage reduction in formazan formation was used as a measure of SOD activity. Catalase measurement (B) was dependent on the ability to catalyze the oxidation of methanol by hydrogen peroxide and the formaldehyde produced was measured colorimetrically. Results are expressed as the mean +/- S.E.M. of three experiments. Asterisks indicate significant differences (*p ≤ 0.05, **p ≤ 0.01) relative to untreated control cells under normal glucose conditions, and triangles indicate significant differences (Δp ≤ 0.05, ΔΔΔp ≤ 0.001) relative to untreated control cells under high glucose conditions.

Superoxide dismutase activity was significantly increased in the presence of high amounts of glucose, which further increased with palmitic acid treatment (Fig 4A). Under normal glucose conditions, 0.06 mM and 0.3 mM palmitic acid caused ~ 45% and 68% increase in SOD activity, respectively. Comparatively, under high glucose conditions, a ~15%-25% increase in SOD activity was observed after palmitic acid treatment. The increase in SOD activity could be due to the increase in superoxide production, which in turn contributed to the increase in ROS production.

Fig 4B shows a concentration-dependent increase in catalase activity under normal and high glucose conditions in the presence of palmitic acid. Furthermore, high glucose significantly increased the catalase activity compared to normal glucose. The increase in catalase activity could clear excess hydrogen peroxide produced by the increase in SOD activity.

### Effect of high glucose/high palmitic acid treatment on GSH metabolism

**Fig 5:**
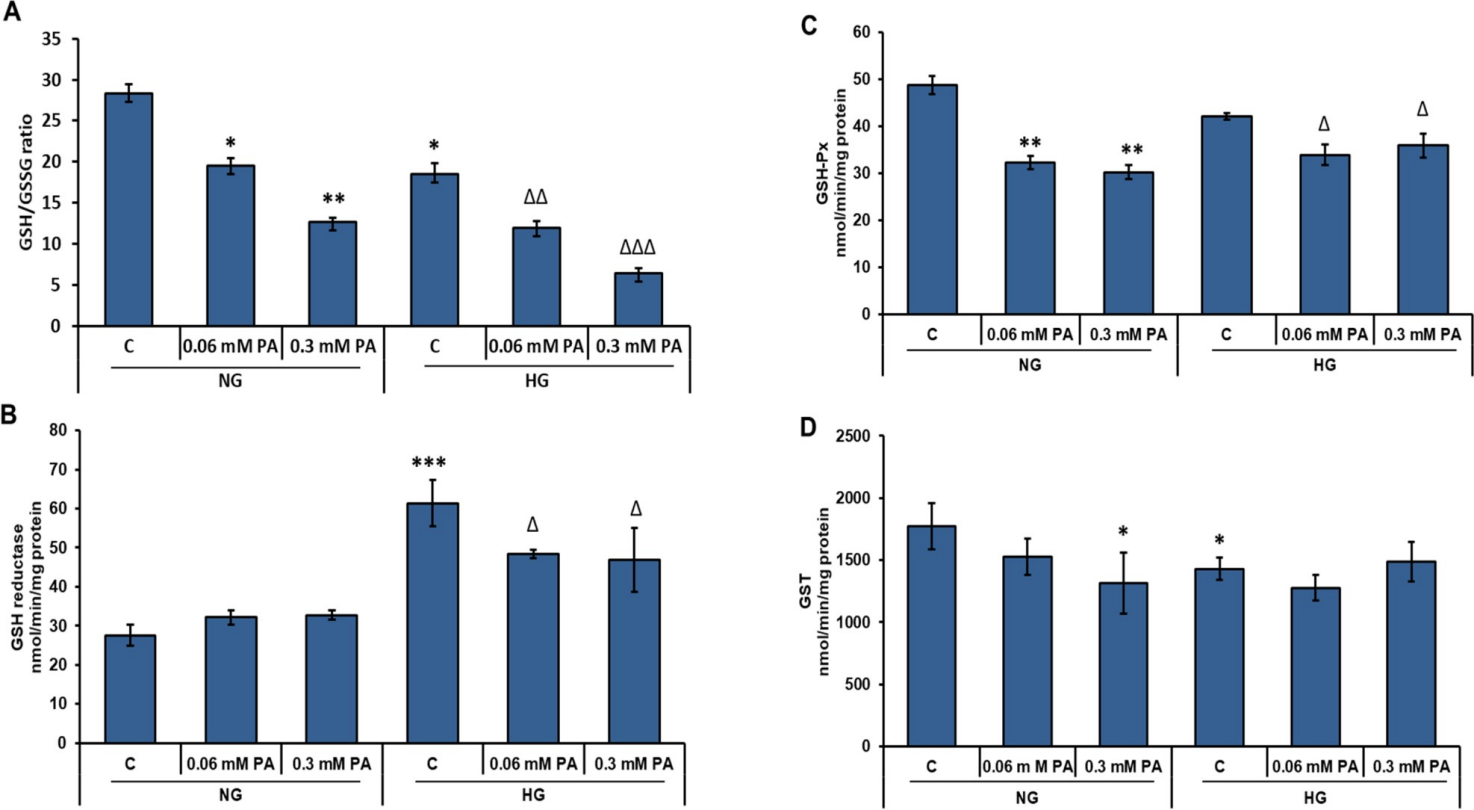
High glucose/high palmitic acid-induced alterations in GSH metabolism. Rin-5F cells were treated with different concentrations of palmitic acid under normal and high glucose conditions and GSH/GSSG ratio (A), GSH reductase (B), GSH-Px (C) and GST (D) were measured as described in the Materials and Methods. Results are expressed as the mean +/- S.E.M. of three experiments. Asterisks indicate significant differences (*p ≤ 0.05, **p ≤ 0.01, ***p ≤ 0.001) relative to untreated control cells under normal glucose conditions, triangles indicate significant differences (Δp ≤0.05, ΔΔp ≤0.01, ΔΔΔp ≤0.001) relative to untreated control cells under high glucose conditions.

Fig 5A shows the inhibitory effect of palmitic acid on the GSH/GSSG ratio, in a concentration-dependent manner, in the presence of normal and high glucose. The inhibitory effect of palmitic acid was more pronounced in the presence of high glucose.

In parallel, the activity of the GSH reductase was significantly decreased with high concentration of palmitic acid in the presence of high glucose (Fig 5B), suggesting a reduction in the recycling mechanism, thus causing an increase in oxidized glutathione. Palmitic acid seemed to have no appreciable effects on GSH-reductase activity in normal glucose-treated cells.

As observed in Fig 5C, palmitic acid evidently reduced the activity of GSH-Px under normal and high glucose conditions. The activity of GSH-conjugating enzyme, GST, slightly decreased with high concentrations of palmitic acid in the presence of normal glucose (Fig 5D). These results may suggest the reduced availability of GSH and a compromised detoxification mechanism in Rin-5F cells treated with high glucose/high palmitic acid.

### Effects of high glucose/high palmitic acid treatment on the release of inflammatory cytokines in Rin-5F cells

**Fig 6:**
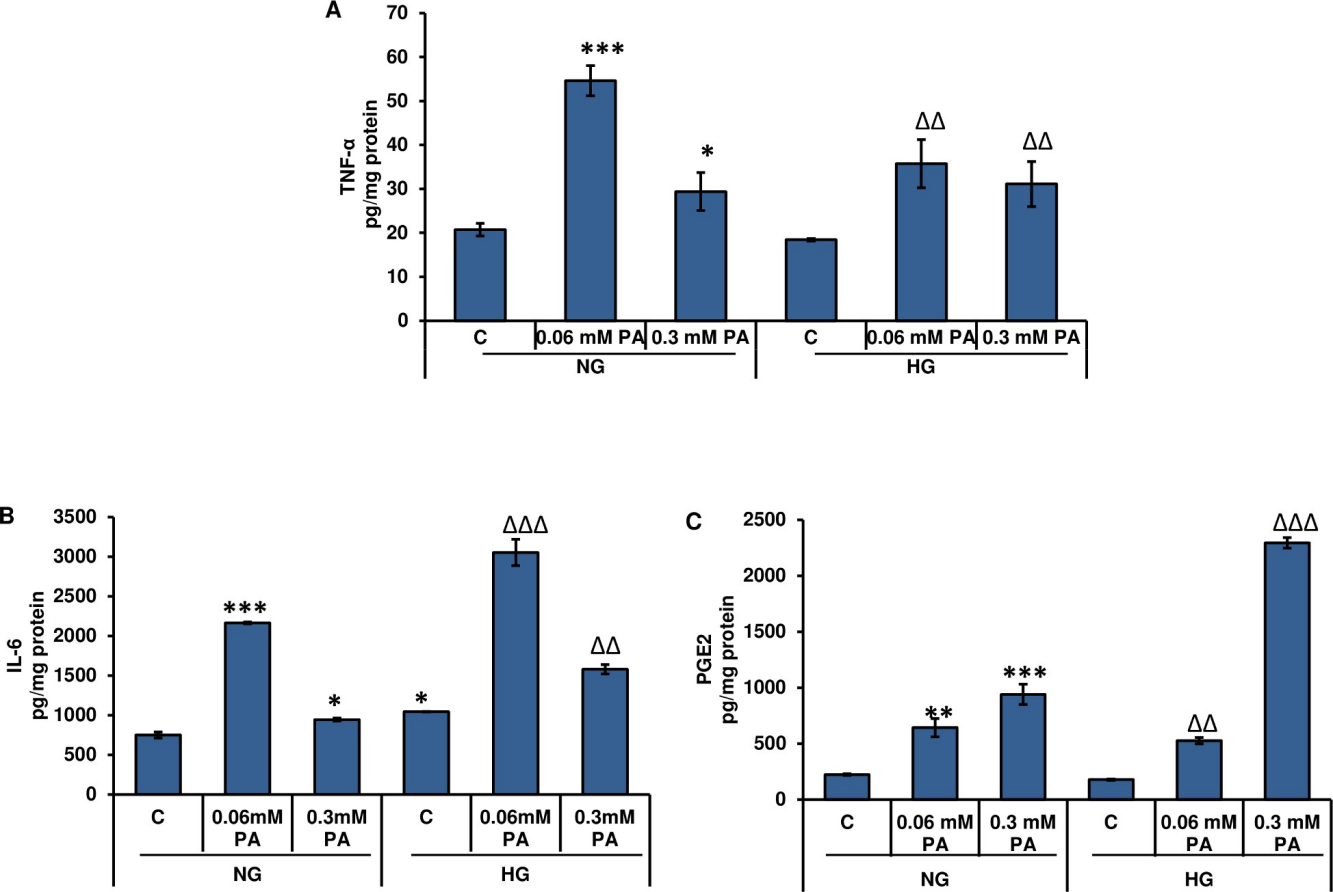
High glucose/high palmitic acid-induced alterations in cytokine levels. Rin-5F cells were cultured to confluence and treated with different concentrations of palmitic acid. TNF-α (A), IL-6 (B) and PGE2 (C) were measured using standard ELISA kits as described earlier in the Materials and Methods. Results are expressed as the mean +/- S.E.M. of three experiments. Asterisks indicate significant differences (*p ≤ 0.05, ** p ≤ 0.01 ***p ≤ 0.001) relative to untreated control cells under normal glucose conditions, triangles indicate significant differences (ΔΔp ≤ 0.01, ΔΔΔp ≤ 0.001) relative to untreated control cells under high glucose conditions.

Fig 6 shows the effect of palmitic acid treatment, under normal and high glucose conditions, on TNF-α and IL-6 levels. A significant increase in the levels of TNF-α and IL-6 were observed with 0.06 mM palmitic acid compared with the 0.3 mM treatment (Fig 6A and 6B). A pronounced increase (approximately 3-fold) in TNF-α levels was observed with 0.06 mM palmitic acid compared with 0.3 mM palmitic acid. However, in the presence of high glucose, the levels of TNF-α increased with both concentrations of palmitic acid. Similar effects were observed with IL-6 levels after palmitic acid treatment. A significant increase was observed with 0.06 mM palmitic acid under normal as well as high glucose conditions. On the other hand, 0.3 mM palmitic acid caused a significant increase only in the presence of high glucose.

Palmitic acid treatment also caused a concentration-dependent increase in prostaglandin E2 (PGE2) levels under both normal and high glucose conditions (Fig 6C).

### Effect of high glucose/high palmitic acid on NF-kB expression

**Fig 7:**
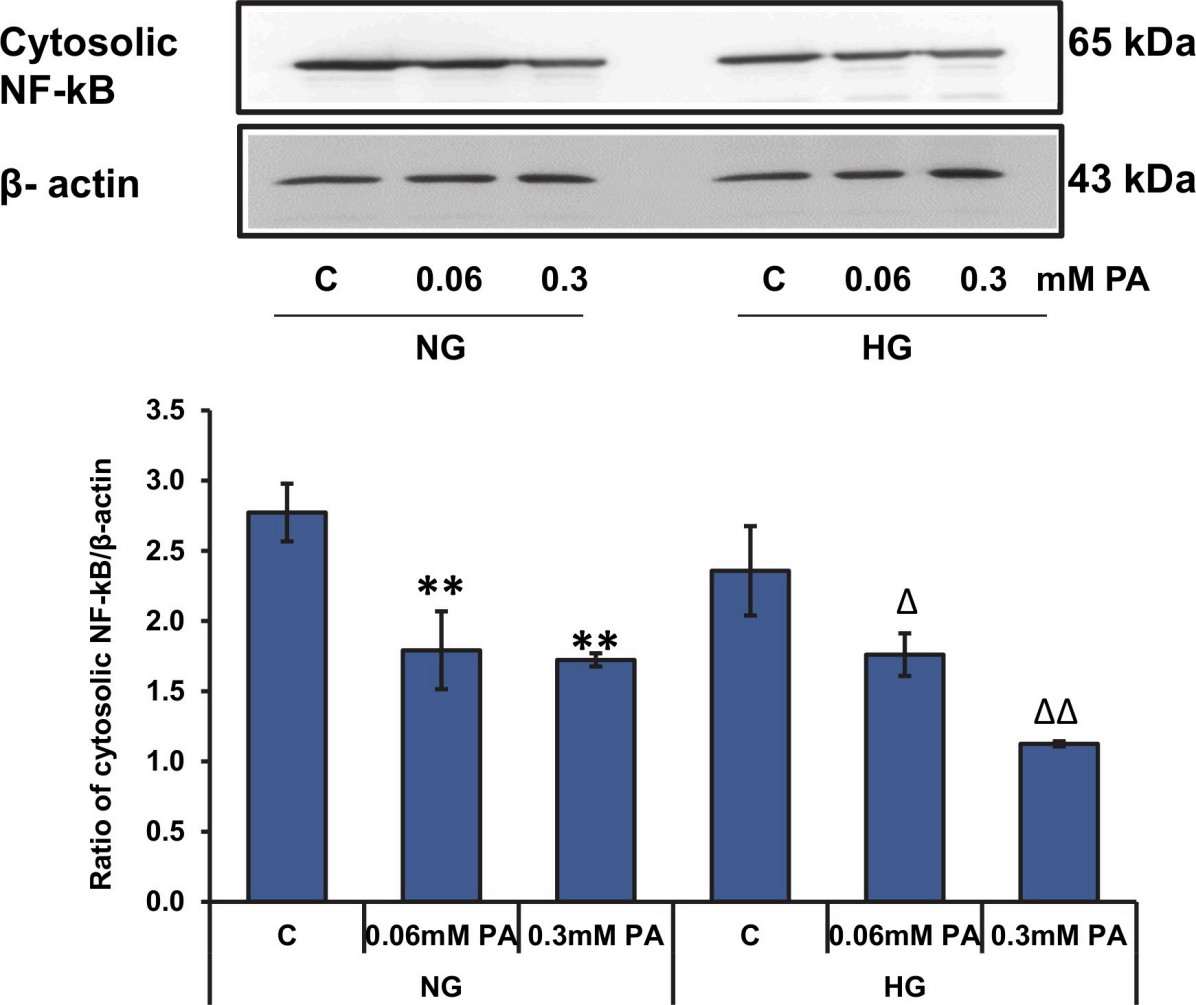
High glucose/high palmitic acid treatment-induced alterations in the expression of NF-κB. Cytosolic extracts (30 μg protein) from treated cells were separated on 7.5% SDS-PAGE and transferred on to nitrocellulose paper by Western blotting. NF-kBp65 protein was detected using a specific polyclonal antibody. The quantitation of the protein is expressed as relative ratios normalized against the loading control, actin. The figures are representative of three experiments. Asterisks indicate significant difference (*p ≤ 0.05, **p ≤ 0.01) relative to untreated control cells under normal glucose conditions, (Δp ≤ 0.05, ΔΔp ≤ 0.01) relative to untreated control cells under high glucose conditions.

As shown in Fig 7, a significant reduction in the expression of cytosolic NF-kB was observed in palmitic acid treated cells suggesting an increased translocation of NF-kB from the cytosol in glucolipotoxicity.

### Effect of NAC pre-treatment on oxidative stress in high glucose/high palmitic acid-treated cells

**Fig 8:**
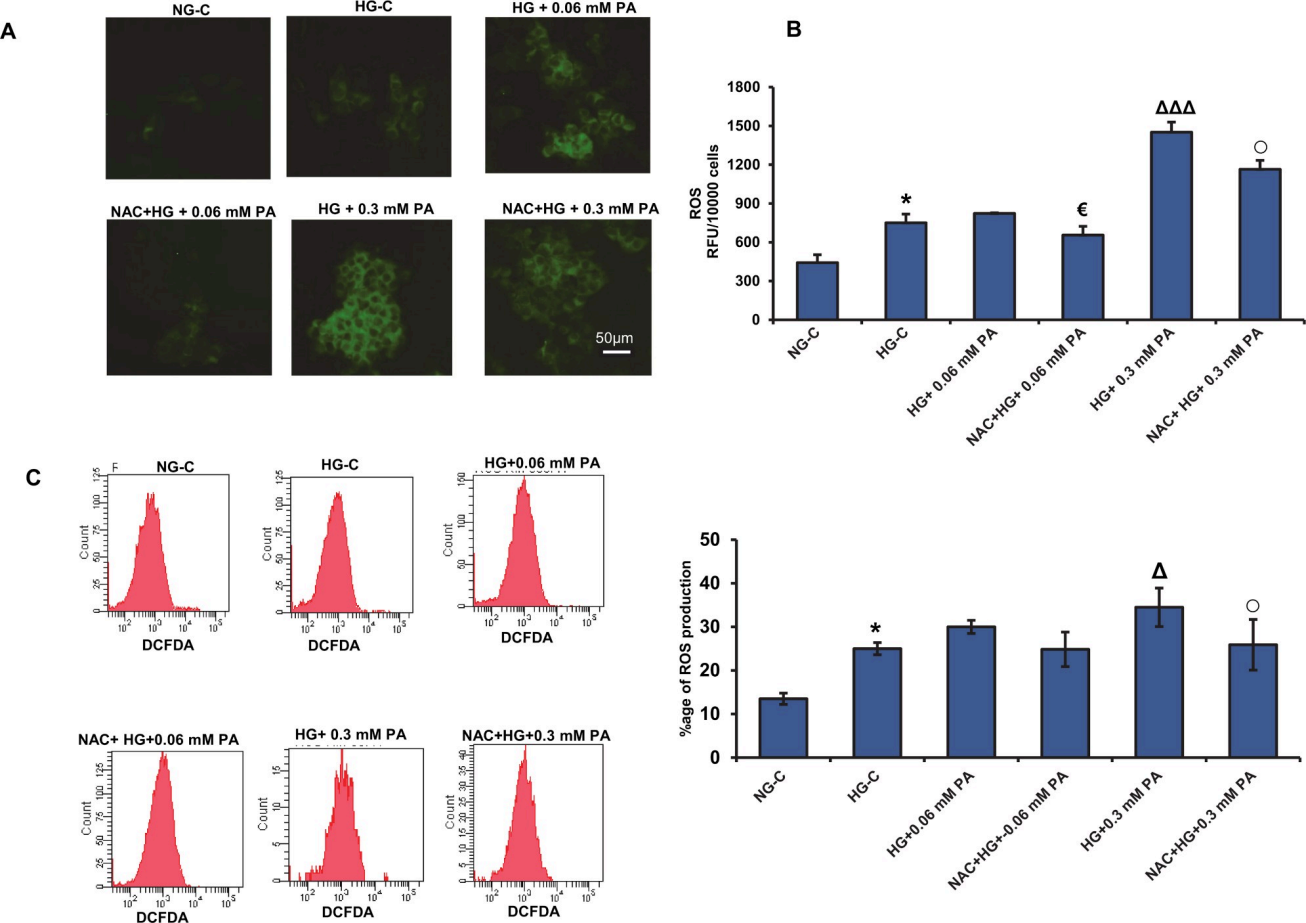
NAC pre-treatment reduced ROS production in high glucose/high palmitic acid-treated Rin-5F cells. Rin-5F cells were grown on cover slips and treated with different concentrations of palmitic acid. Intracellular ROS production was measured using the probe, DCFDA. The fluorescence staining was captured microscopically (A), measured fluorometrically (B) and measured by flow cytometry (C). NAC pre-treatment resulted in a marked reduction in ROS production. Results are expressed as the mean +/- S.E.M. of three experiments. Asterisks indicate significant differences (*p ≤ 0.05) relative to untreated control cells under normal glucose conditions, triangles indicate significant differences (ΔΔΔp ≤ 0.001) relative to untreated control cells under high glucose conditions, (€ p ≤ 0.05) relative to 0.06 mM palmitic acid in the presence of high glucose, (○ p ≤ 0.05) relative to 0.3 mM palmitic acid in the presence of high glucose.

Fig 8 shows the effect of high glucose/high palmitic acid treatment with/without NAC, (10 mM for 2 h), on ROS production in Rin-5F cells. As observed previously, immunofluorescent microscopic, fluorometric and flow cytometric studies showed a significant increase in ROS production with increasing concentrations of palmitic acid, which was significantly reduced (15–25%) in the presence of NAC (Fig 8A, 8B and 8C).

**Fig 9:**
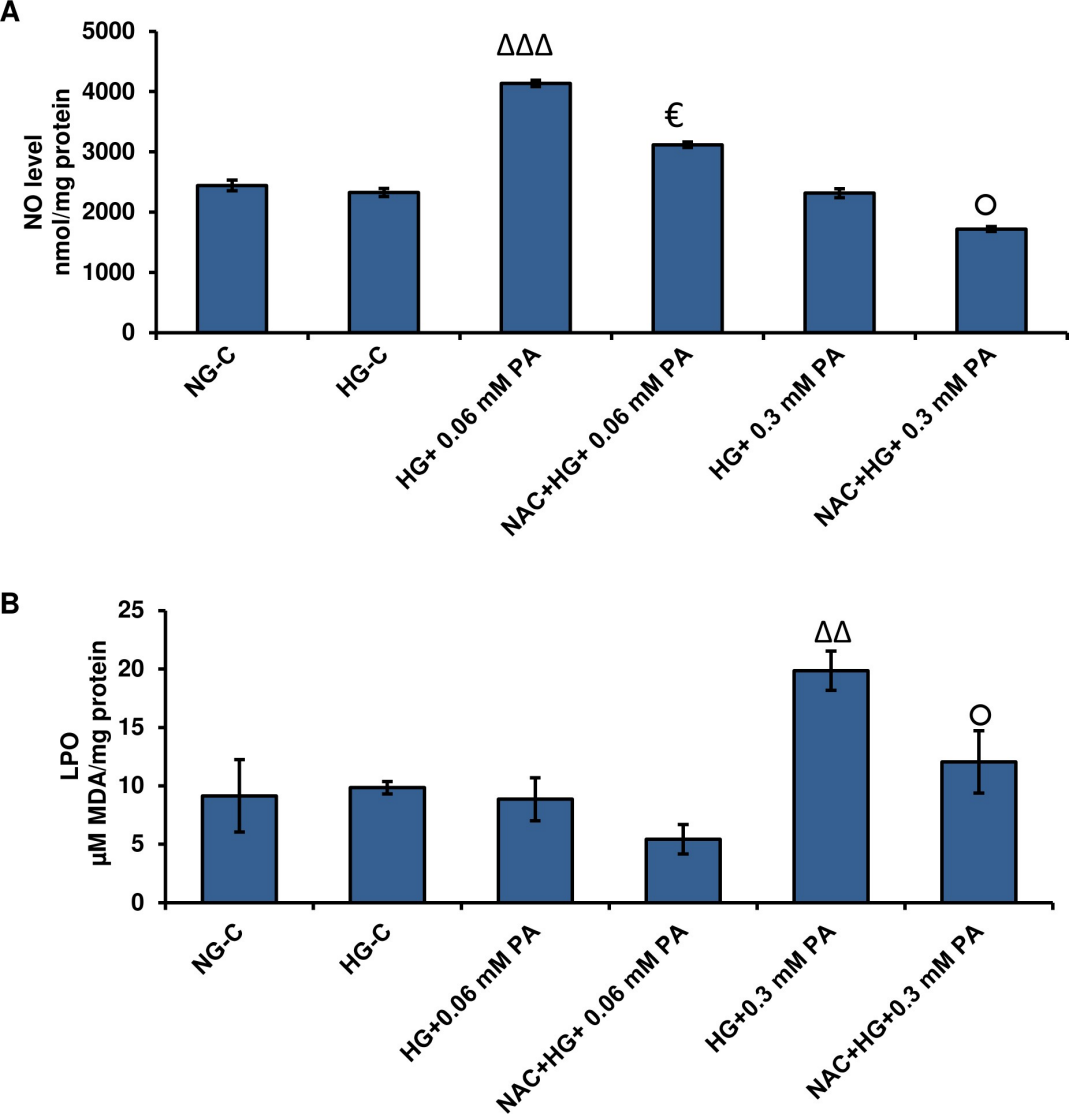
Effect of NAC on NO production and LPO in high glucose/high palmitic acid-induced toxicity in Rin-5F cells. Rin-5F cells were cultured to 70–80% confluence and treated with different concentrations of palmitic acid. NO production was determined by measuring the concentration of total nitrite in culture supernatants (A) and LPO was measured as total amount of malonedialdehyde (B) as per the vendor’s protocol. Results are expressed as the mean +/- S.E.M. of three experiments. Asterisks indicate significant differences (ΔΔp ≤ 0.01, ΔΔΔp ≤ 0.001) relative to untreated control cells under high glucose condition, (€ p ≤ 0.05) relative to 0.06 mM palmitic acid in the presence of high glucose, (○ p ≤ 0.05) relative to 0.3 mM palmitic acid in the presence of high glucose.

As shown in Fig 9A, NAC pre-treatment caused a 20%-25% reduction in NO production with both 0.06 mM and 0.3 mM palmitic acid in the presence of high glucose. Similarly, NAC treatment also caused a significant reduction in LPO, caused by palmitic acid treatment in the presence of high glucose (Fig 9B).

### Effect of NAC pre-treatment on SOD and catalase enzyme activities in high glucose/high palmitic acid-treated cells

**Fig 10:**
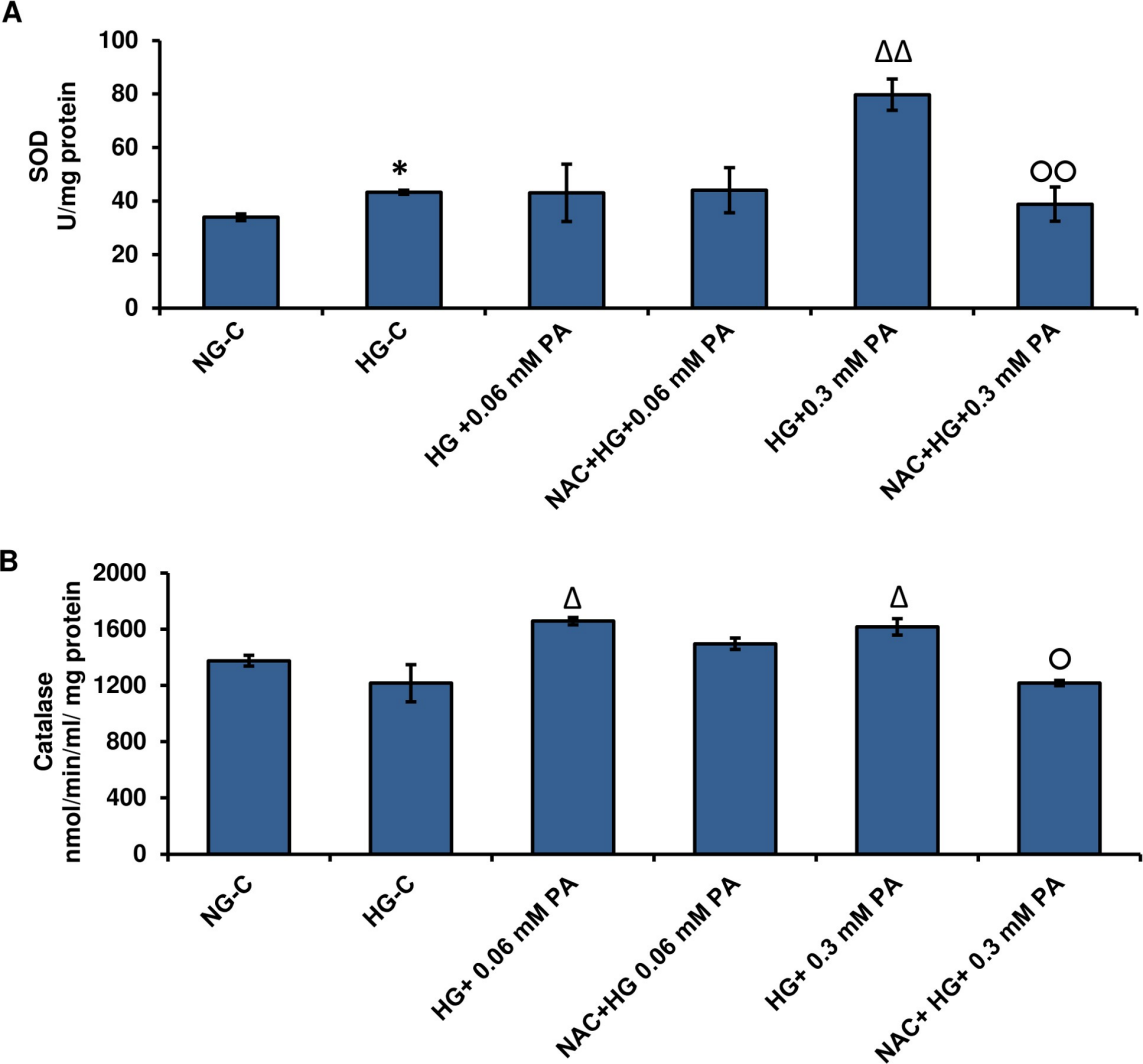
Effect of NAC pre-treatment on SOD and catalase activities in high glucose/high palmitic acid-treated Rin-5F cells. Rin-5F cells were grown to about 80% confluence and treated with different concentrations of palmitic acid with/without NAC. SOD (A) was measured as the percentage conversion of NBT to NBT-diformazan according to the vendor’s protocol. Catalase measurement (B) was dependent on its ability to catalyze the oxidation of alcohol by hydrogen peroxide, and the produced formaldehyde was measured colorimetrically. Results are expressed as the mean +/- S.E.M. of three experiments. Asterisks indicate significant difference (*p ≤ 0.05) relative to untreated control cells under normal glucose conditions, (Δp ≤ 0.05, ΔΔp ≤ 0.01) relative to untreated control cells under high glucose condition, (○p ≤ 0.05, ○○p ≤ 0.01) relative to 0.3 mM palmitic acid in the presence of high glucose.

Pre-treatment with NAC significantly reduced (> 50%) SOD activity, which was increased with 0.3 mM palmitic acid treatment in the presence of high glucose. However, little effect was observed with 0.06 mM palmitic acid (Fig 10A).

Pre-treatment with NAC also caused a mild decrease in catalase activity, which increased with palmitic acid in the presence of high glucose (Fig 10B). These results suggest that the cells were under reduced oxidative stress in the presence of NAC, and a reduction in the concentration of ROS metabolizing enzymes was observed due to reduced ROS production in NAC-treated cells.

### Effect of NAC pre-treatment on GSH levels in high glucose/high palmitic acid-treated cells

**Fig 11:**
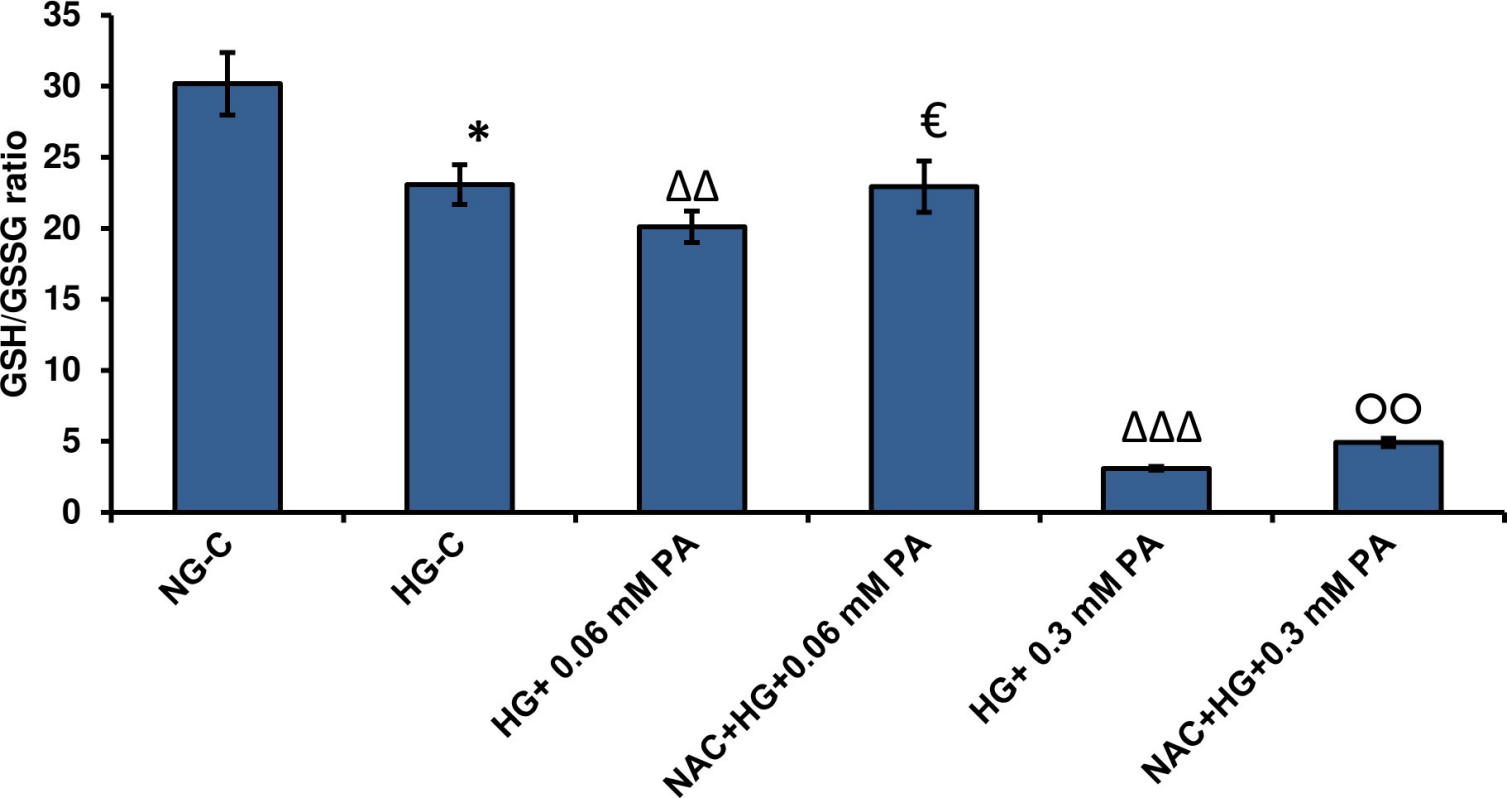
Effect of NAC pre-treatment on GSH levels in high glucose/high palmitic acid-treated Rin-5F cells. GSH/GSSG ratio was measured using a GSH/GSSG-Glo kit as described in Materials and Methods. Results are expressed as the mean +/- S.E.M. of three experiments. Asterisks indicate significant differences (*p ≤ 0.05) relative to untreated control cells under normal glucose conditions, (ΔΔp ≤ 0.01, ΔΔΔp ≤ 0.001) relative to untreated control cells under high glucose conditions, (€p ≤ 0.05) relative to 0.06 mM palmitic acid in the presence of high glucose, (○○p ≤ 0.01) relative to 0.3 mM palmitic acid in the presence of high glucose.

As shown in Fig 11, a significant reduction in GSH/GSSG ratio was observed after high glucose/high palmitic acid treatment. Pre-treatment with NAC markedly enhanced the GSH/GSSG ratio by 20% and 50% after 0.06 mM and 0.3 mM palmitic treatment, respectively.

### Effect of NAC pre-treatment on TNF-α release in high glucose/high palmitic acid-treated cells

**Fig 12:**
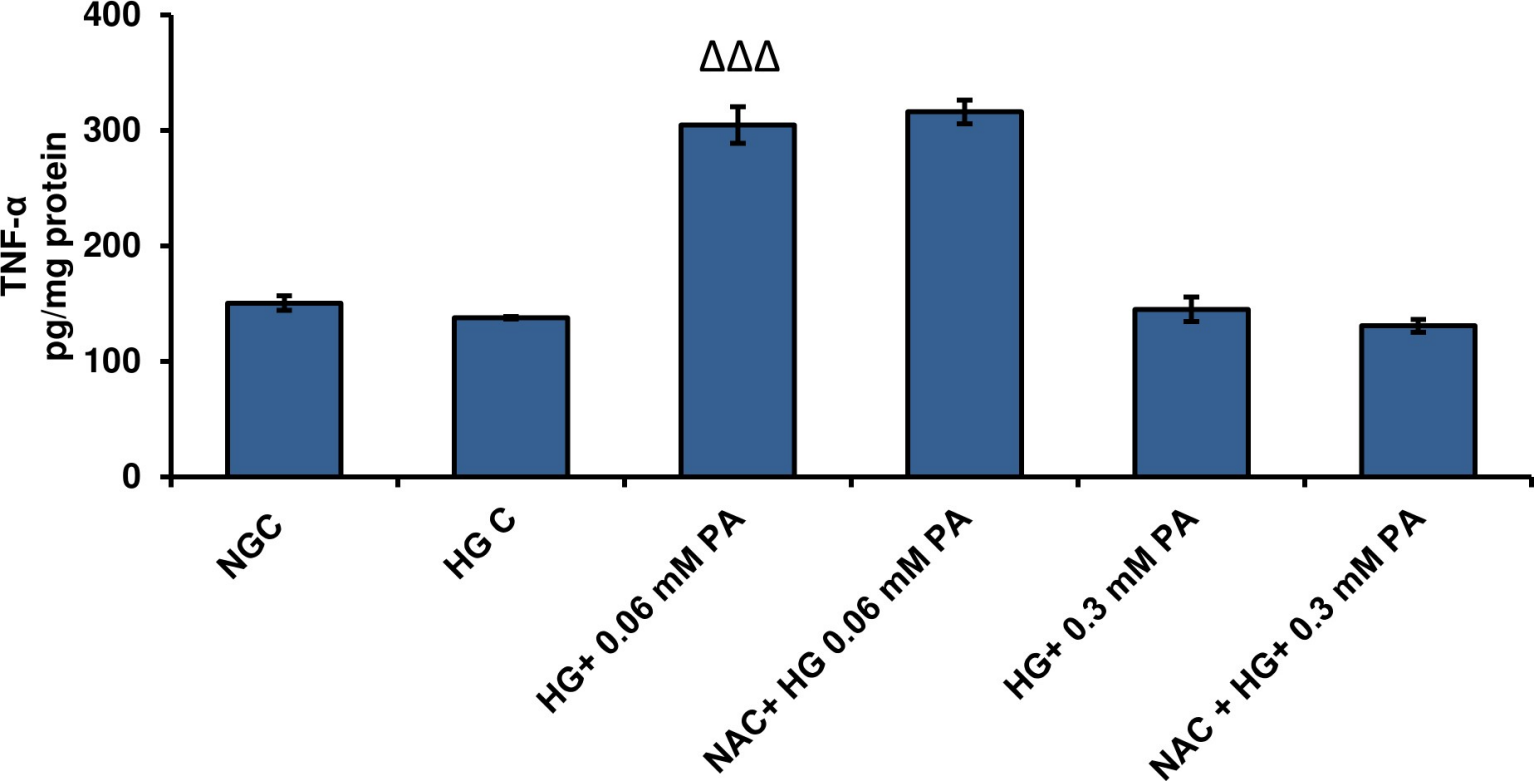
Effect of NAC pre-treatment on the release of TNF-α in high glucose/high palmitic acid-treated Rin-5F cells. TNF-α was measured using standard ELISA kit as described earlier in Materials and Methods. Results are expressed as the mean +/- S.E.M. of three experiments. Triangles indicate significant difference (ΔΔΔp ≤ 0.001) relative to untreated control cells under high glucose conditions.

As shown in Fig 12, TNF-α significantly increased after treatment with 0.06 mM palmitic acid in the presence of high glucose. However, no significant alterations in TNF-α production were observed after NAC pre-treatment.

## Discussion

The concept of glucolipotoxicity has arisen from the combination of deleterious effects of chronic elevation of glucose and free fatty acid levels on pancreatic β-cell function and/or survival [10,11]. Studies have also shown that elevated glucose levels augment the effect of FFA-induced cell death [12]. The precise mechanism of glucolipotoxicity in pancreatic β-cells is still not clear owing to numerous inconsistent reports. Moreover, it is not clearly understood how pancreatic cells adapt to excess fuel and the subsequent fate of cells under such conditions. Nutrient overload induces inflammatory responses causing β-cell mass destruction affecting pancreatic β-cell neogenesis, proliferation and function. Therefore, our aim was to elucidate the cytotoxic mechanisms of high glucose/high palmitic acid treatment in insulin-secreting Rin-5F cells with the main focus on oxidative stress, redox metabolism and inflammatory responses. Furthermore, we also investigated the cytoprotective effects of NAC, a glutathione precursor antioxidant with known ROS scavenging properties, on toxicity induced by high glucose/high palmitic acid in Rin-5F cells.

Pancreatic Rin-5F cells were treated with high glucose (25 mM) and high saturated fatty acid, palmitic acid (up to 0.3 mM). These concentrations were based on previous literature and our preliminary assays on cell viability/survival. The human physiological range for plasma glucose is 5.5–6 mmol/L with a maximum of ∼ 9 mmol/L postprandially [26,27]. Similarly, the physiological range of plasma fatty acids is 0.3 mmol/L to 4 mmol/L [28]. Palmitic acid was selected since it is the most abundant saturated fatty acid [29] and a precursor of several other fatty acids *in vivo*, and has been reported to exert more effects in dyslipidemia when compared to other saturated fatty acids. Moreover, it has been demonstrated clinically, that the level of palmitate in plasma increases 1.5- and 3-fold in type-2 diabetic patients during nocturnal and postprandial states, respectively, compared with a healthy patient [30].

Our results, consistent with other studies [13,31,32], showed that exposure of Rin-5F cells to palmitic acid increased oxidative stress in a concentration-dependent manner under high glucose conditions. This finding is confirmed by an increase in ROS/reactive nitrogen species (RNS) production, increase in lipid peroxidation, inhibition in GSH/GSSG ratio and alterations in GSH metabolism, redox homeostasis and inflammatory response.

The decrease in the activities of GSH-reductase and GSH-Px in the presence of high palmitic acid with normal/high glucose could have triggered the activation of antioxidant enzymes, SOD and catalase. This could indicate the adaptive response of cells to glucose/palmitic acid toxicity. The competency of cells to resist oxidative damage is determined by the effect of a battery of physiological antioxidants, among which GSH is the most abundant. Antioxidant deficiencies could develop as a consequence of either decreased synthesis or increased utilization [33]. Both cases can be explained by a decline in the reduced-to-oxidized GSH ratio. In our study, we observed a significant inhibition of both the recycling enzyme, GSH-reductase, as well as the detoxifying and scavenging enzyme, GSH-Px. It has been well established experimentally and clinically that GSH level is decreased in diabetes. Moreover, GSH deficiency has substantial implications in the pathogenesis of diabetic complications [34–36]. A clinical study has also shown that dietary supplementation of GSH precursors (glycine and glutamine) can restore GSH synthesis and lower the oxidative stress in uncontrolled diabetic patients [34]. Similarly, we observed a significant increase in the GSH/GSSG ratio in high glucose/high palmitic acid-treated Rin-5F cells that have been pre-treated with 10 mM NAC. This resulted in a reduction of oxidative stress in the cells, as observed by a decrease in ROS/RNS production, decreased LPO, and a decrease in antioxidant activity (SOD). This may explain the consequential equilibrium of the redox status provided by NAC that does not require the effects of multiple antioxidant enzymes.

Oxidative stress and inflammation act as cooperative and synergistic partners in the pathophysiology of numerous diseases such as diabetes, obesity, cardiovascular diseases, neurological disorders and cancer. Studies have shown that palmitic acid triggers the production of pro-inflammatory cytokines and oxidants, leading to cellular hypertrophy and apoptosis [37,38]. Fatty acids can directly activate inflammatory pathways themselves, to potentiate inflammatory toxicity. In our study, we observed a decreased expression of cytosolic NF-kB, with increasing palmitic acid concentrations, suggesting an increased translocation into the nucleus, which in turn, triggered the release of pro-inflammatory cytokines such as TNF-α, IL-6 and PGE2.

Additionally, there has been a growing interest in studying the therapeutic effects of N-acetyl cysteine (NAC) in the prevention of diseases characterized by increased oxidative stress, such as diabetes [39]. Therefore, we extended our study to elucidate the effects of NAC pre-treatment under high glucose/high palmitic acid conditions. Our results showed that NAC attenuated the high glucose/high palmitic acid-induced ROS/RNS production and lipid peroxidation and decreased the activities of SOD and catalase. The GSH levels were significantly recovered with NAC pre-treatment. However, NAC pre-treatment did not restore the inflammatory response in pancreatic cells under glucolipotoxicity conditions.

In summary, our results suggest that high glucose/palmitic acid toxicity is partly mediated by increased ROS/RNS production, oxidative stress and inflammatory responses accompanied by alteration of GSH metabolism and an imbalance of redox homeostasis. The NAC treatment, however, has restored most of the redox homeostasis in these cells induced by glucolipotoxicity.

## Supporting information

S1 Fig Checking for mycoplasma. Contamination in the cell-line used was performed using the LookOut mycoplasma detection kit which utilizes the polymerase chain reaction, as described in the vendor’s protocol.
(PDF)

S2 Fig Original Western blot images. Proteins from cell extracts (30 μg) were resolved by 7.5% SDS-PAGE and electrophoretically transferred on to nitrocellulose membranes by Western Blotting. The blots were then developed using an ECL Plus Western Blotting Luminol Reagent kit and the bands visualized using the Typhoon FLA 9500 system (GE Healthcare, Uppsala, Sweden). Actin was used as the loading control. The original blot images after blotting with NF-kB and actin are shown.
(PDF)

## References

[1] Flock MR, Kris-Etherton PM. Diverse physiological effects of long-chain saturated fatty acids: implications for cardiovascular disease. Curr Opin Clin Nutr Metab Care. 2013; 16: 133–140. 10.1097/MCO.0b013e328359e6ac 23037905

[2] Savary S, Trompier D, Andréoletti P, Le Borgne F, Demarquoy J, Lizard G. Fatty acids—induced lipotoxicity and inflammation. Curr Drug Metab. 2012; 13: 1358–1370. 10.2174/138920012803762729 22978392

[3] Masi LN, Rodrigues AC, Curi R. Fatty acids regulation of inflammatory and metabolic genes. Curr Opin Clin Nutr Metab Care. 2013; 16: 418–424. 10.1097/MCO.0b013e32836236df 23739628

[4] Ryu TY, Park J, Scherer PE. Hyperglycemia as a risk factor for cancer progression. Diabetes Metab J. 2014; 38: 330–336. 10.4093/dmj.2014.38.5.330 25349819 PMC4209346

[5] Shimo N, Matsuoka T, Miyatsuka T, Takebe S, Tochino Y, Takahara M, et al Short-term selective alleviation of glucotoxicity and lipotoxicity ameliorates the suppressed expression of key β-cell factors under diabetic conditions. Biochem Biophys Res Commun. 2015; 467: 948–954. 10.1016/j.bbrc.2015.10.038 26471305

[6] Gleason CE, Gonzalez M, Harmon JS, Robertson RP. Determinants of glucose toxicity and its reversibility in the pancreatic islet beta-cell line, HIT-T15. Am J Physiol Endocrinol Metab. 2000; 279: E997–1002. 10.1152/ajpendo.2000.279.5.E997 11052953

[7] Unger RH. Lipotoxicity in the pathogenesis of obesity-dependent NIDDM. Genetic and clinical implications. Diabetes. 1995; 44: 863–870. 10.2337/diab.44.8.863 7621989

[8] Unger RH, Grundy S. Hyperglycaemia as an inducer as well as a consequence of impaired islet cell function and insulin resistance: implications for the management of diabetes. Diabetologia. 1985; 28: 119–121. 10.1007/bf00273856 3888754

[9] Cnop M, Hannaert JC, Hoorens A, Eizirik DL, Pipeleers DG. Inverse relationship between cytotoxicity of free fatty acids in pancreatic islet cells and cellular triglyceride accumulation. Diabetes. 2001; 50: 1771–1777. 10.2337/diabetes.50.8.1771 11473037

[10] Prentki M, Corkey BE. Are the beta-cell signaling molecules malonyl-CoA and cystolic long-chain acyl-CoA implicated in multiple tissue defects of obesity and NIDDM? Diabetes. 1996; 45: 273–283. 10.2337/diab.45.3.273 8593930

[11] Poitout V, Robertson RP. Minireview: secondary β-cell failure in type 2 diabetes-a convergence of glucotoxicity and lipotoxicity. Endocrinology. 2002; 143: 339–342. 10.1210/endo.143.2.8623 11796484

[12] El-Assaad W, Buteau J, Peyot M-L, Nolan C, Roduit R, Hardy S, et al Saturated fatty acids synergize with elevated glucose to cause pancreatic beta-cell death. Endocrinology. 2003; 144: 4154–4163. 10.1210/en.2003-0410 12933690

[13] Alnahdi A, John A, Raza H. Augmentation of Glucotoxicity, Oxidative Stress, Apoptosis and Mitochondrial Dysfunction in HepG2 Cells by Palmitic Acid. Nutrients. 2019; 11: 1979 10.3390/nu11091979 31443411 PMC6770774

[14] Prause M, Christensen DP, Billestrup N, Mandrup-Poulsen T. JNK1 protects against glucolipotoxicity-mediated beta-cell apoptosis. PloS One. 2014; 9: e87067 10.1371/journal.pone.0087067 24475223 PMC3901710

[15] Joshi‐Barve S, Barve SS, Amancherla K, Gobejishvili L, Hill D, Cave M, et al Palmitic acid induces production of proinflammatory cytokine interleukin-8 from hepatocytes. Hepatology. 46: 823–830. 10.1002/hep.21752 17680645

[16] Raza H, Prabu SK, John A, Avadhani NG. Impaired mitochondrial respiratory functions and oxidative stress in streptozotocin-induced diabetic rats. Int J Mol Sci. 2011; 12: 3133–3147. 10.3390/ijms12053133 21686174 PMC3116180

[17] Bradford MM. A rapid and sensitive method for the quantitation of microgram quantities of protein utilizing the principle of protein-dye binding. Anal Biochem. 1976; 72: 248–254. 10.1006/abio.1976.9999 942051

[18] Raza H, John A. Streptozotocin-induced cytotoxicity, oxidative stress and mitochondrial dysfunction in human hepatoma HepG2 cells. Int J Mol Sci. 2012; 13: 5751–5767. 10.3390/ijms13055751 22754329 PMC3382802

[19] Raza H, John A, Shafarin J. NAC attenuates LPS-induced toxicity in aspirin-sensitized mouse macrophages via suppression of oxidative stress and mitochondrial dysfunction. PloS One. 2014; 9: e103379 10.1371/journal.pone.0103379 25075522 PMC4116207

[20] Raza H, Prabu SK, Robin M-A, Avadhani NG. Elevated mitochondrial cytochrome P450 2E1 and glutathione S-transferase A4-4 in streptozotocin-induced diabetic rats: tissue-specific variations and roles in oxidative stress. Diabetes. 2004; 53: 185–194. 10.2337/diabetes.53.1.185 14693714

[21] Habig WH, Pabst MJ, Jakoby WB. Glutathione S-transferases. The first enzymatic step in mercapturic acid formation. J Biol Chem. 1974; 249: 7130–7139. 4436300

[22] Smith IK, Vierheller TL, Thorne CA. Assay of glutathione reductase in crude tissue homogenates using 5, 5’-dithiobis (2-nitrobenzoic acid). Anal Biochem. 1988; 175: 408–413. 10.1016/0003-2697(88)90564-7 3239770

[23] Paglia DE, Valentine WN. Studies on the quantitative and qualitative characterization of erythrocyte glutathione peroxidase. J Lab Clin Med. 1967; 70: 158–169. 6066618

[24] Nahdi AMTA, John A, Raza H. Elucidation of molecular mechanisms of streptozotocin-induced oxidative stress, apoptosis, and mitochondrial dysfunction in Rin-5F pancreatic β-cells. Oxid Med Cell Longev. 2017; 2017: 7054272 10.1155/2017/7054272 28845214 PMC5563420

[25] Al-Nahdi AMT, John A, Raza H. Cytoprotective effects of N-acetyl cysteine on streptozotocin- induced oxidative stress and apoptosis in RIN-5F pancreatic β-cells. Cell Physiol Biochem. 2018; 51: 201–216. 10.1159/000495200 30448838

[26] Nuttall FQ, Ngo A, Gannon MC. Regulation of hepatic glucose production and the role of gluconeogenesis in humans: is the rate of gluconeogenesis constant? Diabetes Metab Res Rev. 2008; 24: 438–458. 10.1002/dmrr.863 18561209

[27] Gerich JE. Control of glycaemia. Baillieres Clin Endocrinol Metab. 1993; 7: 551–586. 10.1016/s0950-351x(05)80207-1 8379904

[28] Abdelmagid SA, Clarke SE, Nielsen DE, Badawi A, El-Sohemy A, Mutch DM, et al Comprehensive profiling of plasma fatty acid concentrations in young healthy Canadian adults. PLoS ONE. 2015; 10 10.1371/journal.pone.0116195 25675440 PMC4326172

[29] Ubhayasekera SJKA, Staaf J, Forslund A, Bergsten P, Bergquist J. Free fatty acid determination in plasma by GC-MS after conversion to Weinreb amides. Anal Bioanal Chem. 2013; 405: 1929–1935. 10.1007/s00216-012-6658-3 23307129

[30] Miles JM, Wooldridge D, Grellner WJ, Windsor S, Isley WL, Klein S, et al Nocturnal and postprandial free fatty acid kinetics in normal and type 2 diabetic subjects: effects of insulin sensitization therapy. Diabetes. 2003; 52: 675–681. 10.2337/diabetes.52.3.675 12606508

[31] Park E-J, Lee AY, Park S, Kim J-H, Cho M-H. Multiple pathways are involved in palmitic acid-induced toxicity. Food Chem Toxicol. 2014; 67: 26–34. 10.1016/j.fct.2014.01.027 24486139

[32] Mangali S, Bhat A, Udumula MP, Dhar I, Sriram D, Dhar A. Inhibition of protein kinase R protects against palmitic acid–induced inflammation, oxidative stress, and apoptosis through the JNK/NF‐kB/NLRP3 pathway in cultured H9C2 cardiomyocytes. J Cell Biochem. 2018; 10.1002/jcb.27643 30259999

[33] Kurutas EB. The importance of antioxidants which play the role in cellular response against oxidative/nitrosative stress: current state. Nutr J. 2016; 15 10.1186/s12937-016-0186-5 27456681 PMC4960740

[34] Sekhar RV, McKay SV, Patel SG, Guthikonda AP, Reddy VT, Balasubramanyam A, et al Glutathione synthesis is diminished in patients with uncontrolled diabetes and restored by dietary supplementation with cysteine and glycine. Diabetes Care. 2011; 34: 162–167. 10.2337/dc10-1006 20929994 PMC3005481

[35] Tan KS, Lee KO, Low KC, Gamage AM, Liu Y, Tan G-YG, et al Glutathione deficiency in type 2 diabetes impairs cytokine responses and control of intracellular bacteria. J Clin Invest. 2012; 122: 2289–2300. 10.1172/JCI57817 22546856 PMC3366396

[36] Hakki Kalkan I, Suher M. The relationship between the level of glutathione, impairment of glucose metabolism and complications of diabetes mellitus. Pak J Med Sci. 2013; 29: 938–942. 10.12669/pjms.294.2859 24353663 PMC3817774

[37] Sergi D, Morris AC, Kahn DE, McLean FH, Hay EA, Kubitz P, et al Palmitic acid triggers inflammatory responses in N42 cultured hypothalamic cells partially via ceramide synthesis but not via TLR4. Nutr Neurosci. 2018; 1–14. 10.1080/1028415X.2018.1501533 30032721

[38] Wang Y, Qian Y, Fang Q, Zhong P, Li W, Wang L, et al Saturated palmitic acid induces myocardial inflammatory injuries through direct binding to TLR4 accessory protein MD2. Nat Commun. 2017; 8 10.1038/ncomms13997 28045026 PMC5216130

[39] Lasram MM, Dhouib IB, Annabi A, El Fazaa S, Gharbi N. A review on the possible molecular mechanism of action of N-acetylcysteine against insulin resistance and type-2 diabetes development. Clin Biochem. 2015; 48: 1200–1208. 10.1016/j.clinbiochem.2015.04.017 25920891

