# Supplementary material for: N-acetyl cysteine attenuates oxidative stress and glutathione-dependent redox imbalance caused by high glucose/high palmitic acid treatment in pancreatic Rin-5F cells
Source: PLoS One. 2019 Dec 20;14(12):e0226696. doi: 10.1371/journal.pone.0226696 (PMC6924679; doi:10.1371/journal.pone.0226696)
Supplement: S1 Fig — Contamination in the cell-line used was performed using the LookOut mycoplasma detection kit which utilizes the polymerase chain reaction, as described in the vendor’s protocol. (PDF) [file pone.0226696.s001.pdf]

S1 Fig. Checking for mycoplasma contamination in the cell-line

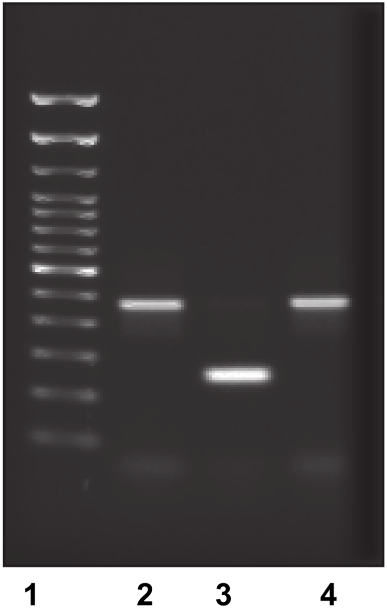

1 - 100 bp ladder
2 - Negative control
3 – Positive control
4 – Cell culture
