# Supplementary material for: N-acetyl cysteine attenuates oxidative stress and glutathione-dependent redox imbalance caused by high glucose/high palmitic acid treatment in pancreatic Rin-5F cells
Source: PLoS One. 2019 Dec 20;14(12):e0226696. doi: 10.1371/journal.pone.0226696 (PMC6924679; doi:10.1371/journal.pone.0226696)
Supplement: S2 Fig — Proteins from cell extracts (30 μg) were resolved by 7.5% SDS-PAGE and electrophoretically transferred on to nitrocellulose membranes by Western Blotting. The blots were then developed using an ECL Plus Western Blotting Luminol Reagent kit and the bands visualized using the Typhoon FLA 9500 system (GE Healthcare, Uppsala, Sweden). Actin was used as the loading control. The original blot images after blotting with NF-kB and actin are shown. (PDF) [file pone.0226696.s002.pdf]

# S2 Fig. Original blots' images

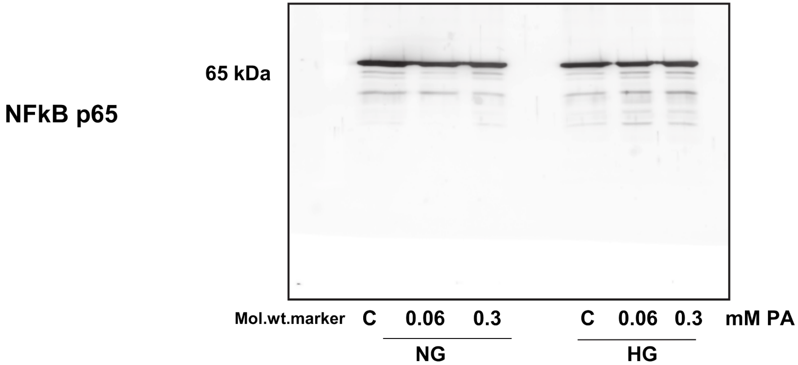


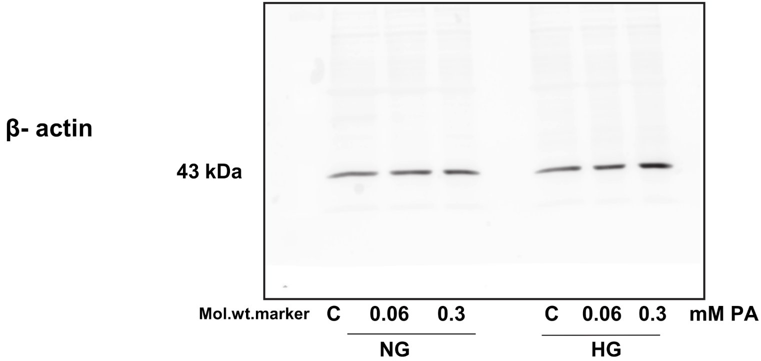


The blots were developed using ECL Plus Western Blotting Luminol Reagent kit and the bands visualized using the Typhoon FLA 9500 system (GE Healthcare, Uppsala, Sweden).
Fig 7 was generated from these blot images.
